# The Holt Ockley System

**Published:** 1909-09-11

**Authors:** 


### Nursing Administration.

### THE HOLT OCKLEY SYSTEM.

### II.—IS IT ECONOMICAL?

We have examined the working of this system from the point of view of the nurses and of the patients. We have seen that the nurse suffers under it in two ways, first, from being imperfectly instructed in her duties, a fact which cannot fail to be of detriment to her in her subsequent career, since in the majority of cases she does not remain a Holt Ockley nurse; secondly, in the circumstances under which she is required to work, residence in the patient's cottage leading to unnecessary hardships in her food and surroundings off duty. Before deciding whether the advantages of the resident nurse overbalance the drawbacks it may be well to consider the financial aspect of the question. Is it more or less of a charge on the patients? Is it more or less lucrative for the nurse? Is it more or less costly to the subscriber?

1. *The Patients.* The rules of the Cottage Benefit Association prescribe that "the services of the nurse must *never* (the italics are not ours) be given gratuitously." It thus becomes apparent that a wide range of cases such as chronics, bad legs, ailing babies, and the like, in which district nurses are able to bring comfort and instruction into the home, are altogether excluded, for poor people are not as a rule able to pay for a resident nurse to tend such cases. "The rate varies according to the class of patient, in some cases ranging as low as a 2s. annual subscription, plus 2s. per week while the nurse is actually employed. Board and lodging for the nurse must in every case be provided by the patient." These are apparently the minimum charges, and reckoning that the nurse's board will cost from 5s. to 7s. a week it results in a charge of from 16s. to 18s. for the fortnight, in addition to the doctor's fee of a guinea, for these nurses are rarely midwives. A district midwife would deliver the mother and give attendance for ten days, night and morning, for 10s. 6d. (in some localities 7s. 6d.), and in addition to her care of the mother during this period would probably visit and advise at intervals for several months previous to the confinement, and keep an eye on the feeding of the baby afterwards. To balance that, the mother would be obliged to get a neighbour or relative to come in for a few hours daily, and for this would probably pay 5s. a week. For cases other than midwifery, there would probably be no charge for a district nurse's services, though an annual subscription would very probably be required as in the case of the Holt Ockley nurse, or a donation for services be encouraged. It is very evident that the district nurse is cheaper for the poor, that she covers a range of work altogether left out of count by the Holt Ockley nurse, and that in so far as she omits certain duties which the Holt Ockley nurse performs, those duties are capable of being satisfactorily undertaken by unskilled women, who are able to learn a good deal from the nurse.

2. *The Nurses.* It is rather difficult to ascertain exactly what the nurses get on this system. In addition to free training, they are paid a salary of from £20 to £25 a year, the patients supply them with board and lodging while they are at cases, and "when out of work the nurse boards herself, lodging being usually provided by the association." The rate of pay is not high, but neither are the qualifications. Their expenses must vary considerably according to the amount of work, and according to the extent to which they find it necessary to supplement from their own purse the board provided.

3. *The Subscribers.* We have before us the report and statement of accounts issued by the Stratton Audley Benefit Nursing Association, a typically well managed branch employing Holt Ockley nurses. It embraces fourteen hamlets, and the number of "benefit subscribers" in these places amounts to a total of 208, the payments varying from 6d. to 10s., with an average of about 2s. Four nurses were employed, whose wages amounted to £108 16s. The number of cases nursed was sixty-three, of which thirty-five were confinements. The report states that "every nurse costs the Association at least 14s. 6d. per week, so that even the fees paid by Classes III. and IV. do not nearly cover the cost of the nurses, and in Classes I. and II. the deficiency amounts to 12s. and 11s. a week." The total expenses of the association nursing these sixty-three cases was £146 7s. 10d., being an average cost of £2 6s. 5d. per case. If it be reckoned that each case lasted on an average a fortnight, as seems to be indicated in the report, a district nurse paying two visits a day would have attended 1,764 times, and at an average cost of 1s. per visit, which few nursing associations exceed, would have cost £88 4s. There is in fact an inevitable waste of nursing material in the system. Two mothers may be confined within a short distance of each other and each will be absorbing the whole time of the nurse. As many as six nurses have been known to be employed in one locality doing work which one midwife could have managed with ease. The loss to subscribers is very considerable. The Cottage Benefit Association takes its stand on the necessity for making all do their part, and determines the status of every person in the parishes within its scope for the purpose of seeing that everyone bears his share. But when it results in charitable subscriptions being required to cover a deficit amounting on an average to over £1 on each case (reckoning their duration at a fortnight) the subscribers may begin to wonder whether after all the work even begins to be self-supporting. Might it not be wiser to leave the patients to arrange about their housework for themselves, a matter they can perhaps do better independently, and provide at a far lower cost that highly specialised aid in sickness which a thoroughly trained nurse alone can afford?

